# European Biomedical Research Infrastructures and the Fight against COVID-19 Pandemic

**DOI:** 10.17691/stm2021.13.1.01

**Published:** 2021-02-28

**Authors:** E.V. Tarasova, P.I. Makarevich, A.Yu. Efimenko, M.A. Kulebyakina, Zh.A. Akopyan

**Affiliations:** Senior Researcher, Department of Scientific Programs and Innovative Technologies, Medical Research and Education Center; Coordinator of the National Contact Point “Health” of the EU Framework Program “Horizon 2020”, Lomonosov Moscow State University, 27/1 Lomonosov Prospect, Moscow, 119192, Russia; Head of the Laboratory of Gene and Cell Therapy, Institute of Regenerative Medicine, Medical Research and Education Center, Associate Professor, Department of Biochemistry and Molecular Medicine, Faculty of Medicine, Lomonosov Moscow State University, 27/1 Lomonosov Prospect, Moscow, 119192, Russia; Head of the Laboratory of Tissue Repair and Regeneration, Institute of Regenerative Medicine, Medical Research and Education Center, Associate Professor, Department of Biochemistry and Molecular Medicine, Faculty of Medicine; Lomonosov Moscow State University, 27/1 Lomonosov Prospect, Moscow, 119192, Russia; Tutor, Department of Biochemistry and Molecular Medicine, Faculty of Medicine, Lomonosov Moscow State University, 27/1 Lomonosov Prospect, Moscow, 119192, Russia; Deputy Director of the Medical Research and Education Center, Head of the Department of Clinical Modeling and Manual Skills, Faculty of Medicine, Lomonosov Moscow State University, 27/1 Lomonosov Prospect, Moscow, 119192, Russia

**Keywords:** EU biomedical research infrastructures, research, innovations, EU, COVID-19, ESFRI

## Abstract

**The study aims** to assess the role of EU biomedical research infrastructures in the fight against the COVID-19 pandemic and to analyze their response to the challenges associated with the spread of the new pathogen.

**Materials and Methods.:**

We analyzed the materials of the Seventh Framework Program for Research and Technological Development (FP7, 2007–2013) of the EU and the Eighth Framework Program “Horizon 2020” (FP8, 2014–2020), official reports of the European Strategic Forum on Research Infrastructures, expert reports, as well as documents of the European Commission, the COVID-19 Data Portal, and other relevant sources of information.

**Results.:**

The analysis revealed that the mechanisms created within the united European research community provided for a flexible response to the emerging threat of COVID-19 as soon as January–May 2020. In particular, information channels were established to timely analyze the research results and coordinate the efforts in the fight against COVID-19. The biomedical infrastructures created in the EU and proved successful earlier have now been mobilized to search for ways of preventing and treating COVID-19. These mechanisms facilitated communication and data exchange between various research institutions and thus laid the ground for new achievements in this area.

**Conclusion.:**

The decisions taken to combat the COVID-19 pandemic have convincingly illustrated that the EU research infrastructures, integrated into a united ecosystem, are highly adaptable and flexible, which allows to realign priorities in a short time and to create instruments that enable scientists to respond to new challenges.

## Introduction

Today, biomedicine as a breakthrough area of modern science requires the creation of special scientific ecosystems. In the XXI century, such systems incorporate scientific instrumentations, databases, biomaterial banks, and specialists capable of providing world-class research.

Research infrastructures (RIs) are complexes of research facilities, resources, and related scientific services that are used by scientists for fundamental and applied research [1]. The RI facilities can be either dispersed, i.e. located in different organizations and/ or countries, or localized in one place, or even virtual. Infrastructures can include scientific equipment, scientific collections, archives, databases, or any objects that can be used for research purposes [2].

The mechanism for the creation and development of a RI has been effectively used by the European Union (EU) to form a united research space. The creation of world-class RIs made it possible to combine the scientific potentials of different countries, and to link individual research tasks with the general purposes of EU development. To develop a unified European strategy for scientific research, the European Strategic Forum for Research Infrastructures (ESFRI) was formed in 2002 [3]. The activities of the forum are aimed at overcoming the fragmentation of efforts of national and regional infrastructures in various research areas, as well as at integrating European the RIs into a global system. As part of its activities, the forum draws up and regularly updates the Roadmap for the development of European infrastructures. In 2009, the European Research Infrastructure Consortium (ERIC) was created to provide these infrastructures with legal status. The ERIC status is assigned to a newly created or existing RI through the European Commission’s approval process, with the infrastructure formally becoming an international organization. If the infrastructure is commercially successful, it becomes possible to create a spin-off company based on this RI, which may increase its economic significance for fundamental and applied science [4].

Currently, European RIs classification includes six thematic domains: 1) energy; 2) environment; 3) health and food; 4) physical sciences and engineering; 5) social and cultural innovation; 6) computing and digital research. Through careful planning, all infrastructures fit seamlessly into the EU scientific landscape, while eliminating duplication of scientific projects and drawing more attention to interdisciplinary research. Due to that, RIs have acquired the flexibility and capability of coherently and rapidly responding to global challenges in a variety of areas. A striking example of this potential was the fight against the COVID-19 pandemic when the EU biomedical infrastructures promptly combined the efforts of experts in virology, epidemiology, and clinical medicine and also attracted scientists from all over the world. As a result, during the COVID-19 pandemic, the research and development process took much less time than that predicted at the beginning.

## Materials and Methods

We analyzed major strategies of the European RI development as outlined in the work programs of the Seventh Framework Program for Research and Technological Development (FP7, 2007–2013), the Eighth Framework Program “Horizon 2020” (FP8, 2014–2020) [5], official reports of the European Strategic Forum on Research Infrastructures, expert group materials, as well as the data of the scientific literature.

The role of RIs in the fight against the pandemic was assessed based on RI information resources, the European Commission portal, and the COVID-19 data portal [6].

## Results

### European research infrastructures in biomedicine.

To identify and monitor majority of the European RIs, the MERIL (Mapping of the European Research Infrastructure Landscape) project was launched in 2014. To date, the project site has turned into an information platform representing the data on more than 1000 infrastructures [7].

More than 300 infrastructures are now registered in the Biological and Medical Sciences section of the MERIL database. Those encompass institutions involved in bioinformatics research (Bioinformatics facilities), cell culturing (Cell culture facilities), translational research (Translational research centers), and over 20 thematic areas of biomedicine.

Biomedical infrastructures include:

facilities and services for medical and clinical research;

biological resource centers;

facilities and services for medical imaging;

omics resources;

bioinformatics resources.

The number of biomedical RIs is steadily growing following the development of biomedicine and the increasing requirements for the quality of scientific research. Thus, the 2006 ESFRI Roadmap included six biomedical RIs; by 2016, their number had doubled [8, 9]. The latest information on biomedical RIs included in the ESFRI roadmap is shown in Table 1.

**Table 1:** Biomedical research infrastructures included in the ESFRI roadmap of 2020 (based on Public Roadmap 2018 Guide [9])

Category infrastructure of research	Name	Mission and goals
Facilities and services for clinical studies	ECRIN-ERIC — European Clinical Research Infrastructure Network	Support for international clinical trials in Europe
	ERINHA — European Research Infrastructure on Highly Pathogenic Agents	Research and development of methods for combating infections caused by risk group 4 pathogens
	EATRIS-ERIC — European Advanced Translational Research Infrastructure in Medicine	Providing support to innovative concepts and their implementations by researchers, companies, and institutions
Biological resource centers	BBMRI-ERIC — Biobanks and Biomolecular Resources	Storage and handling of biological specimens (from sick and healthy individuals) available for information and research purposes
	INFRAFRONTIER — Mouse Archives and Clinics	Provide access to scientific platforms, data, and services for phenotyping, preservation, and dissemination of mouse models for research
	MIRRI — Microbial Resources Research Infrastructure	Development of new biotechnological methods and creation of a united resource space to provide researchers with characterized microorganisms
	EU-OPENSCREEN-ERIC — European Infrastructure of Open Screening Platforms for Chemical Biology	Providing expertise in biological chemistry and pharmaceutics; providing access to a library of chemical compounds and assisting in screening and chemical analysis
Services and resources for medical imaging	Euro-BioImaging — European Research Infrastructure for Imaging Technologies in Biological and Biomedical Sciences	Providing researchers with access to bioimaging technologies and facilities; assistance in data processing and creation of new algorithms for image analysis
Omics resources	Instruct-ERIC — Integrated Structural Biology Infrastructure	Providing access to advanced technologies in structural biology and omics methods
Bioinformatics resources	ELIXIR — A distributed Infrastructure for Life-Science Information	Formation of a platform for the collection, storage, validation, distribution, and use of biological information resources
	ISBE — Infrastructure for Systems Biology	Combining the existing systems biology research infrastructures in Europe and providing researchers with access to new resources, models, and educational programs in the field of bio-modeling

The successful development of European science in recent years indicates advantages of the EU’s approach to planning and creating RIs as a combined project focused on solving global problems. For biomedical RIs, the main goals of researchers and doctors include personalized approaches, transition to digital medicine, and containment of infectious diseases.

The declared goals include not only the transition from the “average patient” model to personalized medicine but also present new requirements for scientific research, reflecting the changing needs and demands of the society. Personalized medicine is a strategy for prevention, diagnosis, and treatment based on molecular genetic characteristics of the body, patient’s lifestyle, and environmental factors. The task of data collection, analysis, and processing is already assigned to European RIs, such as ELIXIR, BBMRI, and INFRAFRONTIER.

The development of new therapeutic strategies requires the accelerated translation of research results and clinical trials, in which the focus shifts from populations to specific groups of patients, and in some studies (case reports) to individual patients. These tasks are performed by the research infrastructures EATRIS and ECRIN, respectively. In addition, accelerating the process of translation requires the use of new bioinformatics methods (ELIXIR), omics techniques (Instruct), screening of new biological substances (EU-OPENSCREEN and Instruct), and an integrated approach to disease modeling (ISBE) using modern technologies for image processing (Euro-BioImaging).

The threat of new infections highlighted by the COVID-19 pandemic, requires the development of innovative medications and vaccines (Instruct). In this respect, the ERINHA program deserves special attention for its emphasis on highly pathogenic microorganisms of risk group 4.

**Figure 1:**
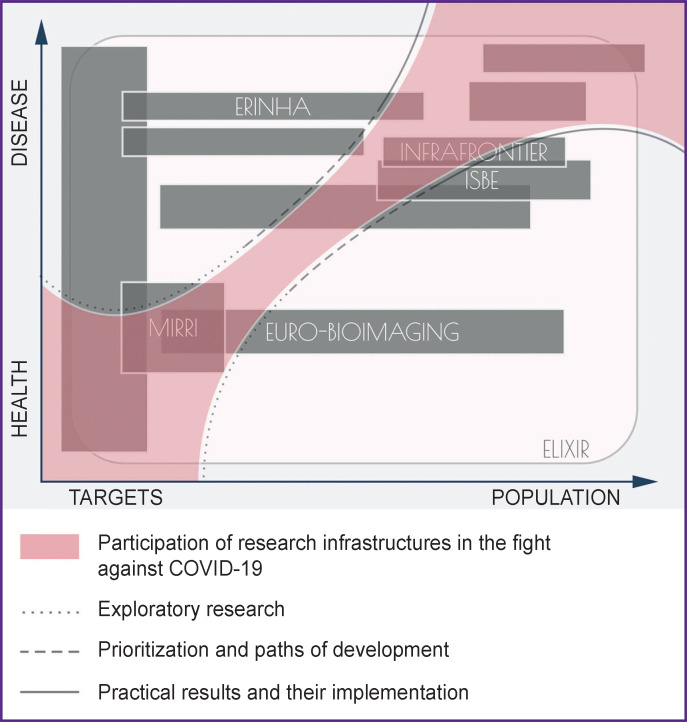
The scientific landscape of EU biomedical infrastructures and their involvement in the fight against the COVID-19 pandemic (authors’ depiction)

By large, all biomedical infrastructures fit into an untied scientific landscape (Figure 1), where they complement each other (while eliminating duplication) in solving a variety of medical problems facing the European scientific community.

Using the map (see Figure 1), researchers can navigate and select the appropriate RI, whereas doctors and healthcare providers can mobilize the resources for conducting applied and translational research.

### Biomedical research infrastructures and COVID-19.

The availability of biomedical RIs and the strict regulation of their activities made it possible in 2020 to quickly respond to the emerging threats of the new coronavirus infection, and effectively use existing institutions and resources to combat the pandemic. Free access to the biomedical RI services has become one of the measures taken by the European Union to counter the emerging challenges (“ERAvsCorona” Action Plan) [10].

Figure 1 schematically shows that all EU biomedical infrastructures, without exception, have joined the fight against the COVID-19 pandemic. Notably, their involvement is not chaotic, but organized in a sequential process, starting from data collection and analysis followed by fundamental research and practical implementation. A key factor for the success of this strategy is the availability of well-developed algorithms for narrowing the search field and selecting priorities for further development. In light of this strategy, the most laborious phases of clinical trials are assisted with methods that have undergone a comprehensive assessment. It becomes possible thanks to bioinformatic approaches, appropriate animal models, and optimal protocols for preclinical and clinical studies.

The EATRIS, ECRIN, and BBMRI were among the first biomedical infrastructures to rise to the challenge caused by COVID-19.

*The European infrastructure for translational research (EATRIS-ERIC — the European Advanced Translational Research Infrastructure in Medicine)* provides support to researchers involved in the development of novel medications and diagnostic methods. An organization that uses EATRIS services on a reimbursable basis receives support at various stages of drug development — from the proof-of-concept stage to preclinical studies — to obtain approval for the first use in humans. This infrastructure includes more than 75 centers (academic institutions and university laboratories), selected for their history and achievements in the development of new drugs and medical technologies. Mainly, EATRIS supports research into gene and cell therapy, biomarkers, low molecular weight (traditional) medications, and vaccines; besides, this RI assists its members in organizing clinical trials.

*European infrastructure network for clinical research (ECRIN-ERIC — European Clinical Research Infrastructure Network)* provides multi-level support for clinical trials. ECRIN provides a wide range of services, including search for funding sources and co-investigators for a successful grant application, selection of an organization for international research monitoring, search for countries and institutions suitable for a clinical study, recruitment of patients, regulatory and ethical expertise related to a clinical trial, provision of relevant documentation; consultations on patient insurance issues, assistance in cost estimation and budgeting related to a clinical trial.

The expansion of knowledge about the molecular mechanisms of diseases, the active development of translational and personalized medicine, the emergence of new methods for the analysis of biological samples put forward ever higher requirements for organizing the work of biobanks. *The European Consortium for Research Infrastructure (BBMRI-ERIC* — *Biobanks and Biomolecular Resources)* is the largest consortium of biobanks in the world. As of 2020, this consortium includes more than 600 biobanks [11] of various categories, types of collections, bio-samples, and storage formats. BBMRI offers various forms of cooperation to its members, including access to a specialized system for the search and exchange of bio-samples, participation in joint research projects, the ability to organize consortia between different biobanks on the BBMRI platform, and many others [12]. This RI aims at harmonizing and standardizing the functioning of biobanks through the creation and implementation of unified standards in bio-banking.

In 2019, these three RIs signed an agreement to create the Alliance for Medical Research Infrastructures, which aims to support close collaboration between translational medicine scientists, biobanks and clinics, and to ensure the exchange of data and biological specimens between their members [13].

Effective management of the research activities was required to adequately respond to the COVID-19 pandemic and to address this issue the Alliance for Medical Research Infrastructures developed recommendations to support the rational use of existing infrastructures [14]. These recommendations set the requirements for the scale and strength of evidence for studies in question, the availability of the appropriate protocols and other information, and the quality management of the studies.

**Figure 2:**
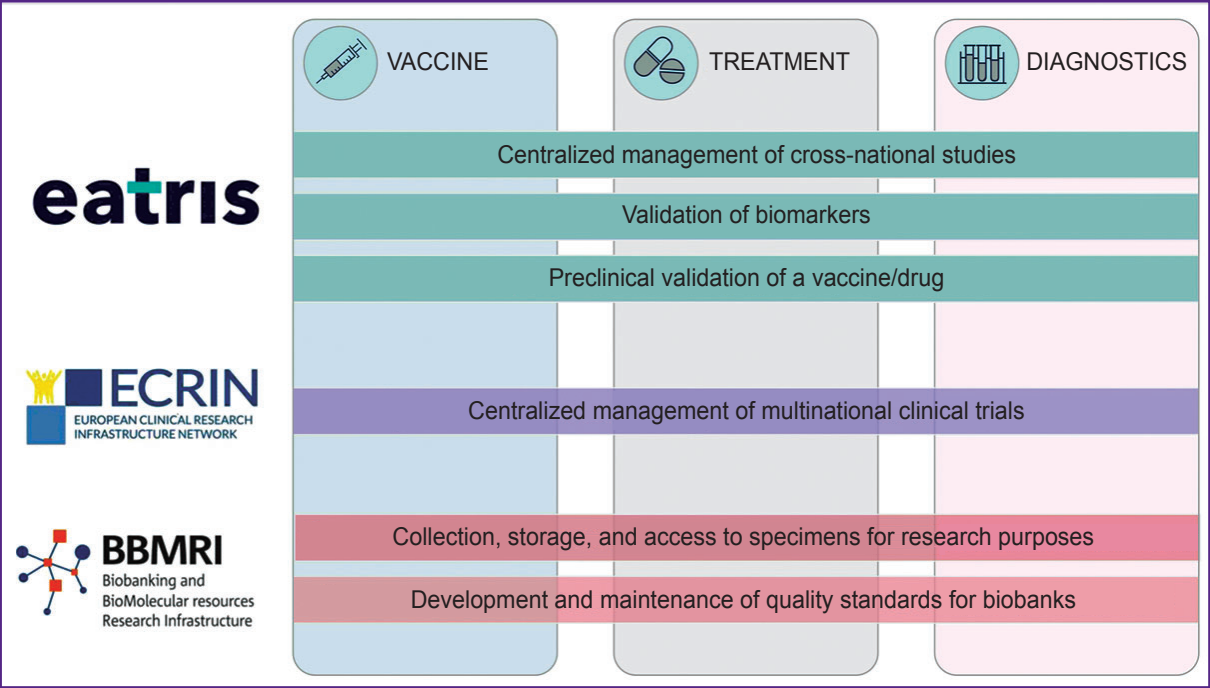
Biomedical activities covered by organizations — members of the Alliance for Medical Research Infrastructures [15]

In addition to the recommendations, the Alliance has published information on the resources each infrastructure provides to combat the pandemic [15], and on the services, each infrastructure can provide for the development of vaccines, drugs, and diagnosis of coronavirus (Figure 2). This information allows researchers and doctors to readily find out which of the three infrastructures can solve a specific biomedical problem.

In addition to EATRIS, BBMRI, and ECRIN, many other biomedical RIs offer their resources to combat the pandemic — from providing access to unique equipment and information to carrying out specific projects on the pathogenesis, diagnosis, and therapy of coronavirus infection, as well as vaccine development. Examples of the unique resources provided by these RIs are given in Table 2.

**Table 2:** Unique resources available at European research infrastructures for combating the COVID-19 pandemic

Name	Resources		Location (clinics, laboratories, etc.)	References
EATRIS-ERIC	Immunological and virological sample testing		iBET — Instituto de Biologia Experimental e Tecnológica University Medical Center Utrecht University of Lisboa	[16]
	High-throughput screening for diagnosis, genotyping, and drug development		IRCCS ISMETT and Fondazione Ri.MED Istituto Nazionale Tumori — IRCCS Fondarione G. Pascale	
	TRANSVAC Initiative — to accelerate the development of new vaccines	Vaccine design and development	University of Lisboa Latvian Biomedical Research and Study Center (BMC) Latvian Institute of Organic Synthesis (IOS) August Pi I Sunyer Biomedical Research Institute (IDIBAPS)	[16, 17]
Instruct-ERIC		Adjuvant development	University of Ljubljana	
ECRIN-ERIC		Testing vaccines in animal models	Biomedical Primate Research Center	
CERIC-ERIC	Studies on the immune response to candidate drugs (SISSI method)		Elettra Sincrotrone Trieste	[18]
	Structural characterization of candidate drugs		TU Graz	
EU-OPENSCREEN-ERIC	Screening and studies on functional characteristics of candidate drugs		Fraunhofer Institute for Molecular Biology and Applied Ecology IME	[19]
ERINHA	High-throughput screening of SARS- CoV-2		Katholieke Universiteit Leuven (KU Leuven) ERASMUS MC	[20]
	Production of viral particles and their quantification			
	Virological studies in animal models			
Instruct-ITALIA	Modeling of molecular structures and protein-ligand interactions		CERM/CIRMMP	[21]
	Analysis of screening results using nuclear magnetic resonance			

Of special note is the contribution of the *European Research Infrastructure on Highly Pathogenic Agents* (*ERINHA*) [22]. This infrastructure brings together European thematic laboratories and national research institutions, coordinating their activities in the area of infectious diseases [23]. The ERINHA experts contributed to solving the tasks related to viral pathogens.

An important step in the fight against COVID-19 is the creation of a vaccine to prevent further spread of coronavirus infection. The task of accelerated vaccine development was assigned to the TRANSVAC2 special project [17] with the participation of EATRIS, Instruct, and ECRIN.

Another important establishment is the *Distributed Infrastructure for Life-Science Information* (*ELIXIR*) responsible for the storage of data on living systems. Their main task is to coordinate and develop European resources used in life science research. Their activities aim to simplify communication between scientists and provide the opportunity to search, analyze and exchange data, expand their expertise and implement the best practices. In the long run, it facilitates the studies on the physiology of living species. ELIXIR’s overall focus is mainly on solving problems related to the “big challenges” facing researchers in the area of life sciences.

An important contribution of ELIXIR was the creation of the *COVID-19 Data Portal* [6] that was incorporated into the joint *European COVID-19 Data Platform*. Using the methods of machine search and data aggregation, the portal collected more than 150 thousand references to studies in the field of virology, infectious diseases, immunology, and clinical medicine. These literature sources provide researchers with quick access to both previous and recent publications. This portal contains publications from as early as the 1940s and includes articles, books, and monographs.

**Figure 3:**
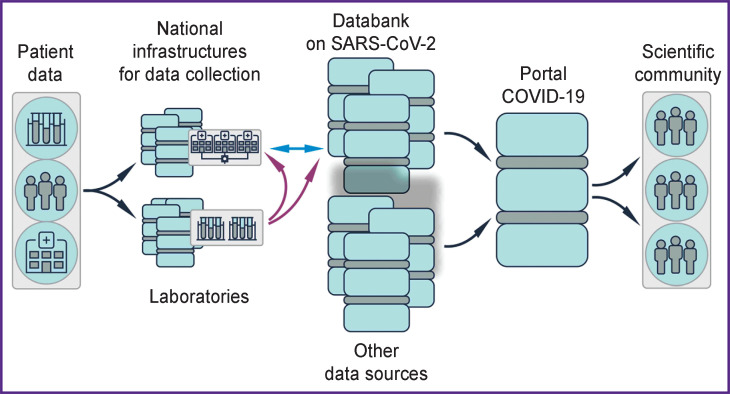
Collection of data and pooling of information for further dissemination through the COVID-19 Data Portal (adapted from [24])

The portal aggregates the information uploaded by researchers to national hubs so to ensure quick and open access to these materials for other scientists. With respect to COVID-19, the most valuable are data of genetic studies (sequences and transcriptome profiles), as well as the structural characteristics of the virus with special attention to the targets for potential antiviral drugs. As a major contribution to the analysis and prognosis of the disease, about 500 genomic sequences of the virus obtained from patients with COVID-19 were posted on the portal in the open-access section (Figure 3).

An equally important step in terms of disseminating scientific information was the launch of the *OpenAIRE COVID-19 Gateway* [25]. In addition to collecting publications, this RI is also tasked with providing the end-to-end navigation through the published reports (articles, software, primary data, research results, protocols, and methods), as well as through data sets related to COVID-19 that had been previously published. By using the “tag” algorithm they created a search engine in which researchers can track the history of a publication from the primary research team to its current status to decide on possible cooperation. As part of the global research integration, other international alliances outside Europe are expected to provide access to their resources.

Another important aspect of the OpenAIRE COVID-19 Gateway is its open access to key publications on COVID-19 that was made possible through cooperation with leading scientific publishers. Currently, the database contains about 95,000 references to publications addressing not only to medical but also to socioeconomic, environmental, and ethical aspects of the COVID-19 pandemic.

### First results produced by biomedical research infrastructures in response to the global challenge posed by the COVID-19 pandemic.

The well-coordinated work of RIs has made it possible to quickly achieve breakthrough results of scientific and medical significance. At the initial stages, methods of systemic and structural biology were used to study the structure of the viral particle. This work was supported and accelerated by the Instruct-ERIC infrastructure. Thus, already in March 2020, a team of researchers led by Martin Walsh described the structure of SARS-CoV-2 serine protease and the spatial organization of its active center [26]. Using these data, it became possible to predict what agents would inactivate the viral protease most effectively and to begin the selection of candidates among potential anti-viral agents. About the same spring of 2020, European researchers established the spatial structures of other SARS-CoV-2 proteins [27], which provided a more complete picture of potential molecular targets in the viral particle.

Thanks to close cooperation between scientists and clinicians, researchers at the Electron Microscopy Department of the European Molecular Biology Laboratory (EMBL) were able, in a few weeks, to characterize the processes developing in human cells infected with SARS-CoV-2. Scientists at the Institute of Biostructures and Bio-imaging (IBB) in Italy investigated the binding of the Spike coronavirus protein to human proteins; these studies resulted in the development of an inhibitor capable of preventing the virus from entering human cells [28].

By April 2020, the primary screening carried out using the resources of the EU-OPENSCREEN infrastructure, made it possible to isolate more than 60 candidate drugs [29]. In the second half of June, it was found that some of the existing drugs were effective in treating patients with coronavirus [30]. By mid-July, as a result of large-scale work supported by ERINHA, data on drug combinations effective against COVID-19 were obtained [31].

Through the effective management of European RIs, measures to prevent the spread of COVID-19 had been developed in the EU in the first few months of the pandemic [32]. Among others, there was a mobile express test that allowed one to diagnose coronavirus infection in half an hour [33].

Significant progress has been made in the development of a vaccine against COVID-19. These efforts were supported by the TRANSVAC initiative, a project aimed to coordinate the work of different RIs (Instruct, EATRIS, and ECRIN) involved in vaccine development. Researchers at the University of Copenhagen participating in the TRANSVAC project reported about a new vaccine against SARS-CoV-2 in early June [34]; a month later, within the framework of this project, preclinical studies of 19 candidate vaccines commenced [35].

## Discussion

Maintaining health as a medical and socioeconomic task requires an integrated approach. The RIs collaborate and complement each other in their contribution to the health care system. The RIs are a proven tool for increasing the efficacy of personalized medicine and globalization of medical care. The RI activities emerged during the COVID-19 pandemic have proved the ability of such infrastructures to effectively respond to social and demographic challenges.

A well-planned infrastructure creates a special research space around itself, including highly professional scientists, effective managers, and technical specialists.

RIs incorporate high-precision scientific equipment, sophisticated installations, and the workforce with the highest level of competence. Assigned with new tasks, they develop their skills based on practical examples, whereas scientists can expand the spectrum of their research, adapt to cooperative work, transparent competition, and ultimately conduct the research more effectively. Thus, a “feedback” system arises, where a scientific problem stimulates the development of a RI, which in turn allows solving new and more complex problems. By interacting with each other, the RI members acquire additional skills that meet the dynamism of the XXI century. Openness, regulated access, and an appropriate organizational structure contribute to the establishment of sustainable and long-term interaction between scientists and research infrastructures, based on both trusting collegial relations and formal obligations of the parties. Ultimately, each RI becomes an important element of the scientific ecosystem and encourages the interaction between its members. The recent events associated with the COVID-19 pandemic have clearly shown the flexibility of RIs and their adaptability to emerging challenges of our time. This vector of development allows scientific institutions to preserve their roles as important components of society and emphasize their significance in solving problems of global health.

## Conclusion

The responses to the COVID-19 pandemic have convincingly illustrated that EU research infrastructures, united into a scientific ecosystem, are highly adaptable and flexible, allowing them to quickly realign priorities, create new tools and provide scientists with unique opportunities to rise to new challenges.
